# Leaf hydraulic vulnerability triggers the decline in stomatal and mesophyll conductance during drought in rice

**DOI:** 10.1093/jxb/ery188

**Published:** 2018-05-18

**Authors:** Xiaoxiao Wang, Tingting Du, Jianliang Huang, Shaobing Peng, Dongliang Xiong

**Affiliations:** 1 National Key Laboratory of Crop Genetic Improvement, MOA Key Laboratory of Crop Ecophysiology and Farming System in the Middle Reaches of the Yangtze River, College of Plant Science and Technology, Huazhong Agricultural University, Wuhan, Hubei, China; 2 Department of Plant Sciences, University of California, Davis, CA, USA

**Keywords:** Drought, leaf hydraulic conductance, mesophyll conductance, photosynthesis limitation, rice, stomatal conductance, vulnerability

## Abstract

Understanding the physiological responses of crops to drought is important for ensuring sustained crop productivity under climate change, which is expected to exacerbate the frequency and intensity of periods of drought. Drought responses involve multiple traits, and the correlations between these traits are poorly understood. Using a variety of techniques, we estimated the changes in gas exchange, leaf hydraulic conductance, and leaf turgor in rice (*Oryza sativa*) in response to both short- and long-term soil drought. We performed a photosynthetic limitation analysis to quantify the contributions of each limiting factor to the resultant overall decrease in photosynthesis during drought. Biomass, leaf area, and leaf width significantly decreased during the 2-week drought treatment, but leaf mass per area and leaf vein density increased. Light-saturated photosynthetic rate declined dramatically during soil drought, mainly due to the decrease in stomatal conductance (*g*_s_) and mesophyll conductance (*g*_m_). Stomatal modeling suggested that the decline in leaf hydraulic conductance explained most of the decrease in stomatal closure during the drought treatment, and may also trigger the drought-related decrease of stomatal conductance and mesophyll conductance. The results of this study provide insight into the regulation of carbon assimilation under drought conditions.

## Teaser

Leaf hydraulic conductance plays a role in stomatal closure during soil drought, and reduction in CO_2_ diffusion is a strong driver of the photosynthetic decline during drought.

## Introduction

Plant productivity is significantly impacted by drought events, which are expected to occur more intensely and frequently as global climate change continues (Trenberth *et al.*, 2013). To develop new approaches to improve crop production under future conditions of water limitation, the responses of several physiological processes, including photosynthesis, plant hydraulic conductivity, and cell turgor pressure, have been widely documented (Flexas *et al.*, 2002; Grassi & Magnani, 2005; Flexas *et al.*, 2009; Galle *et al.*, 2011; Cano *et al.*, 2013; Galmés *et al.*, 2013; Rodriguez-Dominguez *et al.*, 2016; Gleason *et al.*, 2017; Martínez-Vilalta & Garcia-Forner, 2017); however, the correlations among these physiological traits have not been fully evaluated under drought conditions.

In C_3_ plants, the light-saturated leaf photosynthetic rate (*A*) is limited by stomatal conductance (*g*_s_), mesophyll conductance to CO_2_ (*g*_m_), and/or the photosynthetic biochemistry related to either carboxylation velocity, *V*_cmax_, or the maximum electron transport rate set by photochemical and Calvin cycle activities, *J*_max_ (Tosens *et al.*, 2012; Tomás *et al.*, 2013; Tosens *et al.*, 2016; Veromann-Jürgenson *et al.*, 2017). Grassi and Magnani (2005) developed a method to estimate the partial contribution of each limiting factor to the overall reduction of photosynthesis; this approach has since been applied to many species under a variety of environmental stresses (Flexas *et al.*, 2009; Galle *et al.*, 2009; Galle *et al.*, 2011; Galmés *et al.*, 2013; Wang *et al.*, 2018). Although the limiting effects of *g*_s_, *g*_m_, and photosynthetic biochemistry on *A* are dependent on the species, *A* has been suggested to be first inhibited by a decrease in *g*_s_ and *g*_m_ under drought conditions, with the biochemical inhibition occurring later, under more severe drought stress conditions (Grassi & Magnani, 2005; Flexas *et al.*, 2009; Galle *et al.*, 2009; Galle *et al.*, 2011; Galmés *et al.*, 2013; Galmés *et al.*, 2017). However, the contribution of each limiting factor to *A* under drought conditions, especially dynamic drought conditions, is unknown for rice (*Oryza sativa*), despite its status as one of the most important cereal crops in the world.

When plants are exposed to drought, their stomata close, preventing a decline in leaf water potential (ψ_leaf_) and thereby ensuring that the water demand in leaves does not exceed the safe threshold of the hydraulic system (Scoffoni *et al.*, 2017*b*); however, the mechanisms underlying stomatal closure in response to soil drought are poorly understood. Both hormonal (Dodd, 2005) and leaf turgor (Sperry *et al.*, 2002; Buckley, 2005; Brodribb & Cochard, 2009; Rodriguez-Dominguez *et al.*, 2016) signals have been proposed to explain stomatal closure in angiosperms during drought conditions. The hormonal hypothesis suggests that stomatal closure in the leaves is principally driven by hormonal signals, especially abscisic acid (ABA) produced *de novo* in the leaf (Holbrook *et al.*, 2002; McAdam *et al.*, 2016; Zhang *et al.*, 2018). The leaf turgor hypothesis proposes that the decline in *g*_s_ during soil drought is caused by change in leaf turgor. Recently, a serial study (McAdam & Brodribb, 2016; McAdam *et al.*, 2016) tried to link these two hypotheses by demonstrating that, in response to low relative humidity, ABA is rapidly synthesized *de novo* and accumulates in the leaf once the leaf turgor declines in angiosperms. By contrast, a recent theoretical analysis suggested that ABA accumulation in dehydrated leaves is associated with a decline in cell volume, rather than a loss of turgor pressure (Sack *et al.*, 2018).

A decrease in *g*_m_ in response to soil drought was also observed in many previous studies, although the mechanisms for this decrease are unclear (Flexas *et al.*, 2002; Grassi & Magnani, 2005; Warren, 2008; Galle *et al.*, 2009; Cano *et al.*, 2013; Théroux-Rancourt *et al.*, 2014). Many studies have demonstrated the parallel responses of *g*_s_ and *g*_m_ to environmental changes (see review in Flexas *et al.*, 2012). The physiological basis of this relationship is largely unknown; however, recent studies in plant hydraulics suggest that leaf hydraulic conductance (*K*_leaf_) mediates the covariation of *g*_s_ and *g*_m_ (Flexas *et al.*, 2013; Xiong *et al.*, 2015*b*; Xiong *et al.*, 2017; Xiong *et al.*, 2018). The liquid water transport pathways in the mesophyll are partially shared with the CO_2_ diffusion pathways; hence, a functional linkage between *g*_m_ and *K*_leaf_ has been suggested. Similarly, *g*_s_ and *K*_leaf_ may be coupled because of the common stomatal pathway for the exchange of water and CO_2_ between the leaf and the atmosphere. Correlations between *K*_leaf_ and *g*_s_ or *g*_m_ have been observed in many species and genotypes (Brodribb *et al.*, 2005; Brodribb *et al.*, 2007; Flexas *et al.*, 2013; Théroux-Rancourt *et al.*, 2015; Xiong *et al.*, 2018). Nonetheless, it is unclear whether a coordinated regulation of *g*_s_, *g*_m,_ and *K*_leaf_ occurs under varied environmental conditions, for instance, during water stress. Indeed, *K*_leaf_ declines rapidly between full turgor and the turgor loss point and even more strongly during extreme dehydration (reviewed in Scoffoni *et al.*, 2017*b*). The response of *K*_leaf_ to dehydration has been suggested to arise mainly due to the vulnerability of tissues outside the xylem, such as mesophyll (Scoffoni *et al.*, 2017*a*), the major tissue where water transport and CO_2_ diffusion may share a common pathway (Xiong *et al.*, 2017). Théroux-Rancourt *et al.* (2014) observed that *K*_leaf_ and *g*_s_ decreased as the soil water potential declined, but that *g*_m_ decreased only after *g*_s_ was <0.15 mol m^−2^ s^−1^ in poplars (*Populus* sp.). Revealing the regulatory patterns of these traits in response to drought is necessary for enhancing our understanding of plant responses to water limitation (Scoffoni *et al.*, 2017*b*).

In this study, we estimated gas exchange, *K*_leaf_, and leaf turgor in response to both short- and long-term soil drought in two rice genotypes to reveal the correlations between and sequences of changes in these traits during the response to drought stress. The objectives of this study were (i) to reveal the dynamic limiting effects of *g*_s_, *g*_m_, and the photosynthetic biochemistry on *A* during drought in rice; and (ii) to clarify the vulnerabilities of *A*, *g*_s_, *g*_m_, and *K*_leaf_ and their relationships under drought conditions.

## Materials and methods

### Plant materials and growth conditions

**Fig. 1.**
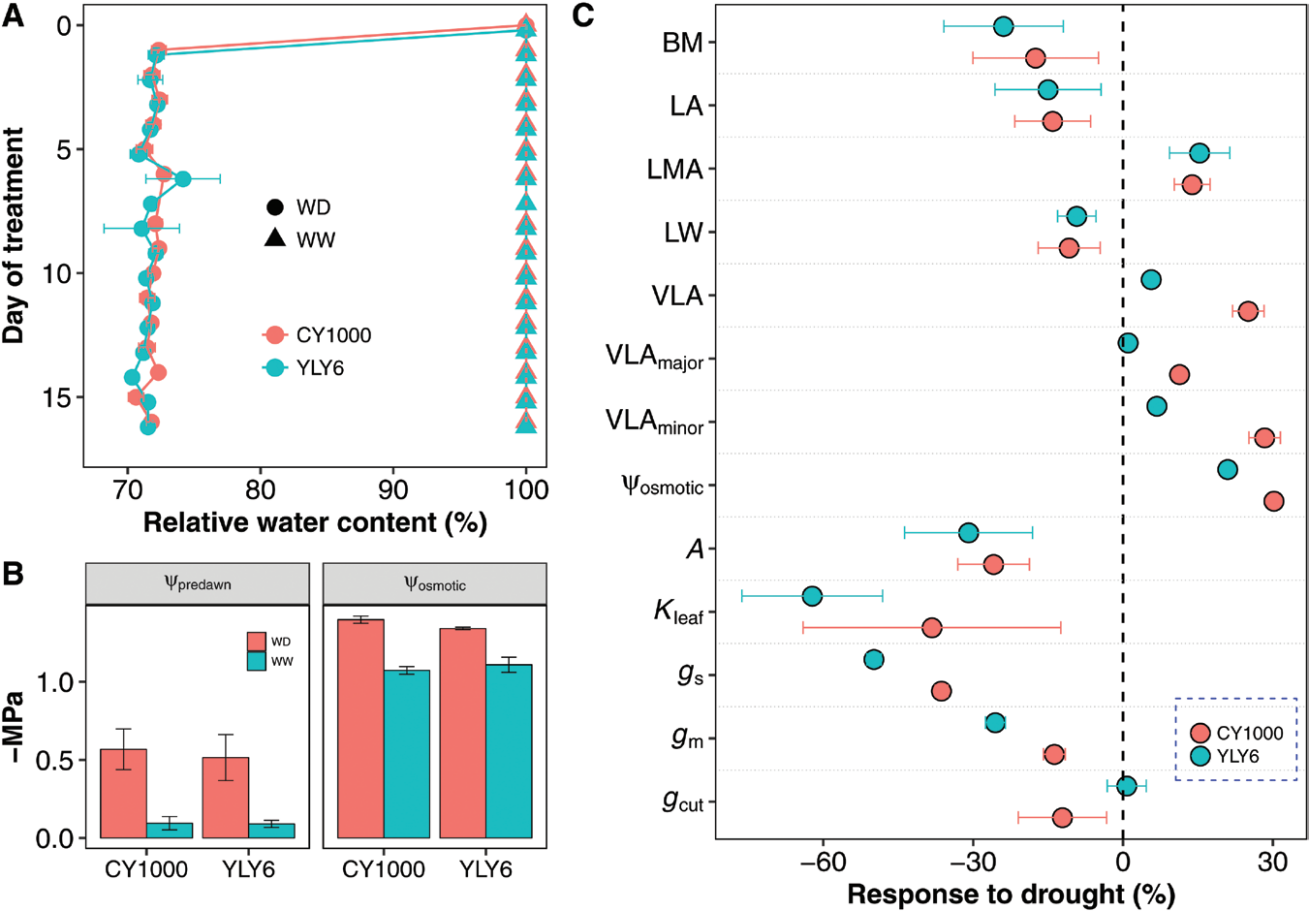
(A) Soil relative water content of the well-watered (WW) and water-deficient (WD) treatments. (B) Predawn leaf water potential (ψ_predawn_) and leaf osmotic potential (ψ_osmotic_) of two rice genotypes after a 2-week drought treatment. See Supplementary Table S1 for definitions of the parameters. (C) Responses of leaf traits to 2 weeks of drought. The response was calculated as ln(*X*_WD_/*X*_WW_), where *X*_WD_ and *X*_WW_ were mean values of trait *X* under the WD and WW treatments, respectively.

Two ‘Super’ hybrid rice cultivars, Yangliangyou 6 (YLY6) and Chaoyou 1000 (CY1000), were used in this study. YLY6 is a widely used reference cultivar in promotion trials of newly developed ‘Super’ varieties in China, while CY1000 is a recently developed ‘Super’ variety with high yield and wide adaptation characteristics. Seeds were germinated and grown in a nursery for 2 weeks, and the seedlings were then transplanted into 11 litre plastic pots containing 10 kg of soil, at a density of three plants per pot. Before transplantation, 7.0 g of compound fertilizer (N:P_2_O_5_:K_2_O=16:16:16%; Batian Ecological Engineering Limited, Shenzhen, China) was mixed into the soil of each pot. For each genotype, 60 randomly arranged pots of seedlings were grown, and pots were watered daily before the drought experiment began. Seven weeks after transplantation, 10 pots of each genotype were subjected to long-term water deficiency stress by maintaining a relative soil water content of ~75% for 2 weeks (Fig. 1A).

### Gas exchange and chlorophyll fluorescence measurements

To avoid the effects of fluctuations in outdoor air temperatures, light intensity, and humidity (see Supplementary Fig. S1 at *JXB* online) on gas exchange, each measurement was taken between 09.00 h and 16.00 h in an environmentally controlled growth chamber (Model GR48; Conviron, Controlled Environments Limited, Winnipeg, MB, Canada), with an air temperature of 25 °C, a relative air humidity of 70%, and a photosynthetic photon flux density (PPFD) of 600 μmol m^−2^ s^−1^. The night before the gas exchange measurements, the second fully expanded leaf of each plant was covered with both a plastic sheet and aluminum foil to estimate the stem water potential (ψ_stem_) of the plant. After acclimating the plants overnight in the growth chamber, gas exchange measurements were carried out on the uppermost newly and fully expanded leaves, using a LI-6400 portable photosynthesis system equipped with a LI-6400–40 chamber (LI-COR Inc., Lincoln, NE, USA). In the leaf chamber, the PPFD was maintained at 1500 μmol m^−2^ s^−1^, the leaf-to-air vapor pressure deficit (VPD) was 1.5–2.0 kPa, and the CO_2_ concentration was adjusted to 400 μmol mol^−1^ using a CO_2_ mixer. The block temperature during the measurements was set to 25 °C. After stabilization to a steady state, the gas exchange parameters, steady-state fluorescence (*F*_s_), and maximum fluorescence (*F*′_m_) were recorded. The actual photochemical efficiency of photosystem II (Φ_PSII_) was calculated as follows:

$$\Phi_{\text{PSII}}\text{=}\frac{\left(F_{\text{m}}^{`}-F_{\text{s}}\right)}{F_{\text{m}}^{`}}$$ (1)

The electron transport rates (*J*_f_) were computed as follows:

$$J_{\text{f}}=\Phi_{\text{PSII}}\cdot \text{PPFD}\cdot \alpha \cdot \beta$$ (2)

where *α* is the leaf absorbance and *β* represents the distribution of electrons between photosystem I and photosystem II. After the gas exchange measurement, both ψ_stem_ and the leaf water potential (ψ_leaf_) were determined using a pressure chamber (PMS Instrument Company, Albany, OR, USA) after equilibrating for at least 30 min.

To estimate *α* and *β*, light response curves for both well-watered and water-stressed plants were measured. The gas exchange system was switched to a low O_2_ concentration (<2%) by injecting pure N_2_, and simultaneous measurements of the light response curves and chlorophyll fluorescence were performed. During the measurements, the chamber conditions were the same as those described above, except that a gradient of PPFD values was used: 2000, 1500, 1200, 1000, 800, 600, 400, 200, 100, and 0 µmol m^−2^ s^−1^. After reaching a steady state, the parameters of gas exchange and chlorophyll fluorescence were simultaneously recorded. The slope of the relationship between Φ_PSII_ and 4Φ_CO2_ (the quantum efficiency of CO_2_ uptake) was considered to represent the value of *α·β* (Valentini *et al.*, 1995). As there were no differences in the *α·β* values between the control and water-stressed leaves, the average value for all genotypes was used.

The mesophyll conductance of CO_2_ (*g*_m_) was calculated based on the variable *J* method described by Harley *et al.* (1992), as follows:

$$g_{\text{m}}=A/\text{(}C_{\text{i}}-\frac{\Gamma *\left(J_{\text{f}}+\text{8}\left(A+R_{\text{d}}\right)\right)}{J_{\text{f}}-\text{4}\left(A+R_{\text{d}}\right)}\text{)}$$ (3)

where Γ* represents the CO_2_ compensation point in the absence of respiration, *R*_d_ is the day respiration rate, which was assumed to be half of the dark respiration rate (*R*_dark_), *C*_i_ represents the intercellular CO_2_ concentration, which was determined from an estimation of the cuticular conductance (see below) in this study, and *C*_c_ is the CO_2_ concentration in the chloroplast. Г* is related to the Rubisco specific factor (*S*_C/O_), which is relatively conserved at a given temperature. In the present study, the rice *S*_C/O_ at 25 °C was obtained from Hermida-Carrera *et al.* (2016).

### Cuticular conductance and *C*_i_ calibration

The method of Sack and Scoffoni (2011) was used to estimate the minimum leaf conductance (*g*_cut_). The leaves were scanned using a Canon EOS M50 (Canon Inc., Tokyo, Japan) to calculate their area, and then dried in a room with an air temperature of 25.0 °C and a light intensity of <5 µmol m^−2^ s^−1^. Leaves were weighed every 10 min over ~300 min using a digital balance (Sartorius BP 2215, Gottingen, Germany). The cuticular transpiration rate was determined from the regression of the change in leaf mass over time. Temperature and humidity sensors (HOB; H21-002; Onset Computer Corporation, Bourne, MA, USA) were placed next to the samples, and the air temperature and relative humidity were recorded at the beginning of each weighing cycle to determine the VPD. The value of *g*_cut_ was calculated as the transpiration rate divided by the VPD.

It is a widely accepted norm that water vapor diffusing through stomata can be used to calculate the *C*_i_; however, the calculations assume an identical gas phase path for CO_2_ and water vapor, which does not hold under drought conditions. As stomata close, the cuticle becomes the dominant path of water vapor diffusion (Boyer *et al.*, 1997; Boyer, 2015*a*; Hanson *et al.*, 2016). Indeed, it has been suggested that *C*_i_ could potentially be overestimated, as the cuticular conductance is far greater for water than for CO_2_ (Boyer, 2015*b*). Thus, in this study, we recalculated *C*_i_ to take *g*_cut_ into account, as follows:

$$C_{\text{i}}=\frac{\text{(}g_{\text{sc}}\text{-}E_{s}/2\text{)}\cdot C_{as}-A}{g_{\text{sc}}\text{+}E_{s}/2}$$ (4)

$$g_{\text{sc}}=\text{(}g_{\text{lw}}\text{-}g_{\text{cut}}\text{)}/1.6$$ (5)

$$E_{\text{s}}=E_{\text{l}}-g_{\text{cut}}(W_{\text{l}}-W_{a})$$ (6)

where *C*_as_ is the CO_2_ concentration at the leaf surface (400 µmol mol^−1^), *g*_sc_ is the true stomatal conductance to CO_2_, *E*_s_ is stomatal transpiration, *g*_lw_ is the total leaf conductance to water, *E*_l_ is leaf transpiration, and *W*_l_ and *W*_a_ are the water vapor values inside and outside the leaf, respectively.

### Hydraulic vulnerability

Three methods were used to estimate *K*_leaf_: the standard evaporative flux method (EFM), the rehydration kinetic method (RKM), and the gas exchange-based EFM method. The EFM was calculated following the methods outlined by Scoffoni *et al.* (2012) and Xiong *et al.* (2017). The rice tillers were bench-dried, and then the initial leaf water potential (ψ_0_) was measured in the neighboring leaves. The dehydrated leaves were excised from the tillers under water and connected to a tube system, which was connected to a reservoir of degassed water situated on a high-precision digital balance (NBL 84e, Adam Equipment Inc., Oxford, UK). The balance logs data to a computer every 10 s. Once the water flow rate was stable (~30 min), the water flow rate (*E*) into the leaves under favorable conditions (on a large box fan with PPFD>1000 µmol m^−2^ s^−1^) was recorded, along with the leaf temperature. Next, the leaf area (LA) and final leaf water potential (ψ_final_) were measured. The *K*_leaf-EFM_ was calculated as follows:

$$K_{\text{leaf-EFM}}=\frac{E}{\text{LA}\cdot {\text{(0-ψ}}_{\text{final}}\text{)}}$$ (7)

The RKM was calculated following the method outlined by Blackman and Brodribb (2011). The ψ_0_, leaf temperature, initial maximum rehydration flow of water into leaves (*I*), and LA were measured in a similar manner to the EFM method described above, except that the leaves were covered with moist paper and were not exposed to light, in order to prevent transpiration during the *I* measurement. *I* was calculated by fitting an exponential curve through the first 10 s of the flow data and extrapolating back to the initial point of leaf excision. *K*_leaf-RKM_ was calculated as follows:

$$K_{\text{leaf-RKM}}=\frac{I}{\text{LA}\cdot {\text{ψ}}_{0}}$$ (8)

We also measured *K*_leaf_ using the transpiration rate (*T*_r_) values from the gas exchange measurement and ψ_stem_, and ψ_leaf_ after the gas exchange. For this method, *K*_leaf-licor_ was calculated as follows:

$$K_{\text{leaf-licor}}=\frac{T_{\text{r}}}{{\text{ψ}}_{\text{stem}}-{\text{ψ}}_{\text{leaf}}}$$ (9)

To construct the vulnerability curve, *K*_leaf_ was plotted against the lowest ψ_leaf_ (i.e. ψ_0_ in RKM; ψ_0_ or ψ_final_ in EFM, and ψ_leaf_ in the gas exchange method). Because the viscosity of water is temperature dependent, the *K*_leaf_ values in this study were standardized to their corresponding value at 25 °C (Scoffoni *et al.*, 2012).

### Pressure–volume curves

Four pressure–volume curves per genotype were conducted with well-watered plants, to estimate their osmotic potential at full turgor (π_0_) and at the turgor loss point (π_tlp_), as well as their modulus of elasticity (*ɛ*) (Sack *et al.*, 2003; Scoffoni *et al.*, 2011). Leaves were sampled from well-watered plants and rehydrated overnight before desiccation. Briefly, the leaf weight and ψ_leaf_ were measured at least 10 times over the desiccation period until ψ_leaf_ dropped to –3.0 MPa. Finally, the leaves were dried at 70 °C for 2 days and their dry mass was measured.

### Osmotic potential measurements

The fully expanded young leaves of well-watered and water-stressed plants were sampled in the morning. The leaf samples were immersed in liquid nitrogen and then stored at –80 °C. The osmotic potentials of these leaves were measured using a vapor pressure osmometer (VAPRO 5520; Wescor Inc., Logan, UT, USA).

### Leaf vein density

The newly developed and fully expanded leaves of both well-watered and water-stressed plants were chemically cleared in 15% NaOH (w/v) and then bleached following the standard protocol for rice (Xiong *et al.*, 2015*b*; Xiong *et al.*, 2017). The cleared leaves were stained with safranin and fast green in ethanol. After being rinsed in water, the leaves were scanned using a Canon EOS M50 (Canon Inc., Tokyo, Japan) to enable quantification of their area and major vein lengths. To measure the minor veins, a light microscope (U-TVO.5XC; Olympus, Tokyo, Japan) with a 5× objective was used to observe the leaves, and photographs were taken of the top, middle, and bottom of each leaf. LA and vein length were manually measured using ImageJ (Wayne Rasband/NIH, Bethesda, MD, USA). The total vein density (VLA), major vein density (VLA_major_, including the midrib and large veins), and minor vein density (VLA_minor_) were estimated.

### Biomass and leaf area

Four plants per treatment were sampled after 2 weeks of drought treatment and were separated into stems and leaves. The LA was measured using a LA meter (LI-3100; LI-COR Inc., Lincoln, NE, USA). The samples were dried to a constant weight at 80 °C and their biomass was recorded.

### Photosynthetic limitation analysis

A limitation analysis is a helpful tool for quantifying the effects of stress on various factors affecting *A* (Grassi & Magnani, 2005; Buckley & Diaz-Espejo, 2015). The relative photosynthetic limitations, including the relative stomatal (*l*_s_), mesophyll (*l*_m_), and biochemical (*l*_b_) limiting effects, were modeled as previously described by Grassi and Magnani (2005):

$$l_{\text{s}}=\frac{g_{t}\text{/}g_{\text{sc}}\cdot \partial A\text{/}\partial C_{\text{c}}}{g_{t}+\partial A\text{/}\partial C_{\text{c}}}$$ (10)

$$l_{\text{m}}=\frac{g_{t}\text{/}g_{\text{m}}\cdot \partial A\text{/}\partial C_{\text{c}}}{g_{t}+\partial A\text{/}\partial C_{\text{c}}}$$ (11)

$$l_{\text{b}}=\frac{g_{t}}{g_{t}+\partial A\text{/}\partial C_{\text{c}}}$$ (12)

where *g*_t_ is the total conductance, which is calculated as:

$$g_{\text{t}}=\frac{1}{\frac{1}{g_{\text{sc}}}+\frac{1}{g_{\text{m}}}}$$ (13)

To assess the impact of ψ_leaf_ change on photosynthesis, the limiting effects were linked to overall changes in *A*:

$$\frac{dA}{A}=\text{LS+LM+LB}=\frac{dg_{\text{sc}}}{g_{\text{sc}}}l_{\text{s}}+\frac{dg_{\text{m}}}{g_{\text{m}}}l_{\text{m}}+\frac{dJ_{\text{f}}}{J_{\text{f}}}l_{\text{b}}$$ (14)

where LS, LM, and LB are the reduction fractional limitations in *A* caused by a reduction in stomatal conductance, mesophyll conductance, and biochemistry, respectively. In the current study, the fitted photosynthetic parameters at ψ_leaf_ = –0.3 MPa were used as reference values. Thus,

$$\frac{dx}{x}≅\frac{x_{0.3}-x}{x_{0.3}}$$ (15)

where *x* represents the fitted *g*_s_, *g*_m_, or *J*_f_, (see Supplementary Table S1 for definitions of these and other mathematical parameters used in this paper), and *x*_0.3_ represents the *x* value at ψ_leaf_ = –0.3 MPa.

### Quantification of the contributions of hydraulic and hormonal signals to stomatal closure

The g_s_ model, originally presented by Buckley *et al.* (2003) and subsequently modified by Rodriguez-Dominguez *et al.* (2016), was used to examine the contributions of hydraulic and hormonal signals to the decline in *g*_s_ under drought. In this model, *g*_s_ is expressed as:

$$g_{\text{s}}=\frac{naK_{\text{leaf}}(\psi_{stem}+\pi )}{K_{\text{leaf}}+na\Delta w}$$ (16)

where *π* is bulk leaf osmotic pressure, Δ*w* is the leaf-to-air water vapor mole fraction gradient, *n* represents the effect of hormonal signals on the sensitivity of guard cell osmotic pressure to leaf turgor, and *a* represents the relative adenosine triphosphate concentration. In this study, *K*_leaf_, ψ_stem_, Δ*w*, and *π* were measured, *a* was simulated, and *n* was fitted. The *π* value was measured using a WP4C water potential meter (Decagon, Pullman, WA, USA).

### Statistical analysis

Regressions were fitted with a linear model, and regression lines are shown when *P*<0.05. The correlations between the leaf functional traits (*K*_leaf_, *A*, *g*_s_, *g*_m_, and *J*_f_) and ψ_leaf_ were tested by four functions described in a previous study (Scoffoni *et al.*, 2012): a linear function (*K*_leaf_ = *a*ψ_leaf_+*b*), a sigmoidal function ($K_{\text{leaf}}=\frac{a}{\text{1+}e^{\text{-(}\frac{{\text{ψ}}_{\text{leaf}}-x_{0}}{b}\text{)}}}$), a logistic function ($K_{\text{leaf}}=\frac{a}{\text{1+(}\frac{{\text{ψ}}_{\text{leaf}}}{x_{0}}{\text{)}}^{b}}$), and an exponential function ($K_{\text{leaf}}=y_{0}+a\cdot e^{\text{-}b\cdot {\text{ψ}}_{\text{leaf}}}$). The functions were compared using the Akaike Information Criterion (AIC) corrected for low *n*. The function with the lowest AIC value was chosen as the maximum likelihood function. The differences of *K*_leaf_ vulnerability among genotypes and methods were compared using a two-sample Kolmogorov–Smirnov test. All of the analyses were performed in the program R (R Core Team, 2018).

## Results

### Effects of 2 weeks of water stress on plant performance

The 2-week drought treatment led to a significant reduction in rice biomass (by 17.5% in CY1000 and 20.9% in YLY6; Fig. 1). Drought stress significantly increased LMA (by 13.8% in CY1000 and 15.5% in YLY6) and VLA (by 25.1% in CY1000 and 5.7% in YLY6), but decreased LA (by 14.1% in CY1000 and 15.0% in YLY6) and leaf width (LW) (by 10.8% in CY1000 and 9.3% in YLY6). VLA_major_ increased by 11.33% in CY1000 under water stress, but no changes were observed in YLY6; more pronounced increases in VLA_minor_ were observed for both genotypes (28.4% increase in CY1000 and 6.8% in YLY6).

Drought stress significantly decreased the gas exchange and leaf hydraulic traits in both rice genotypes, with more pronounced effects in YLY6 than CY1000 (Fig. 1). Overall, water stress decreased *A*, *g*_s_, *g*_m_, and *K*_leaf_ by 28.4%, 43.0%, 19.6%, and 50.2%, respectively. The leaf osmotic potential (ψ_osmotic_) increased by 30.2% in CY1000 and 21.0% in YLY6 following the 2-week drought treatment. In addition, water stress decreased *g*_cut_ in CY1000, but not in YLY6 (Fig. 1).

### Leaf hydraulic and photosynthetic dynamics during short-term drought

**Fig. 2.**
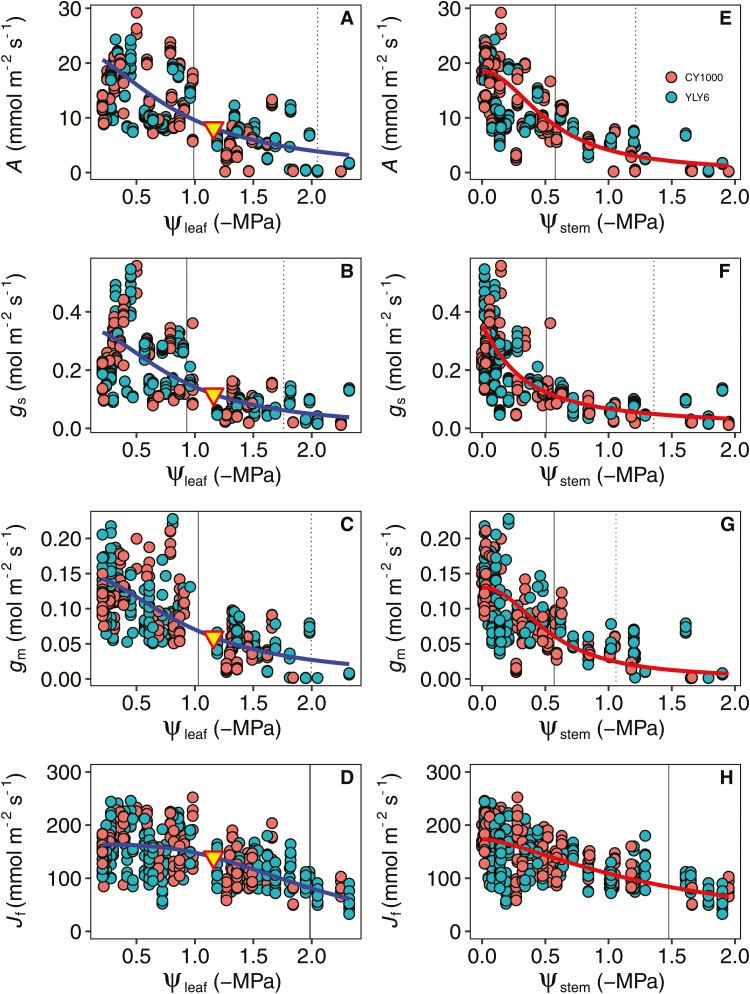
Response of the gas exchange parameters to decreasing (A–D) leaf water potentials (ψ_leaf_) and (E–H) stem water potentials (ψ_stem_). The vertical solid and dotted lines indicate the water potential at 50% and 80% loss of function, respectively. The triangle represents the turgor loss point (Table 1). Fitted lines are the best-fit functions selected using maximum likelihood.

**Fig. 3.**
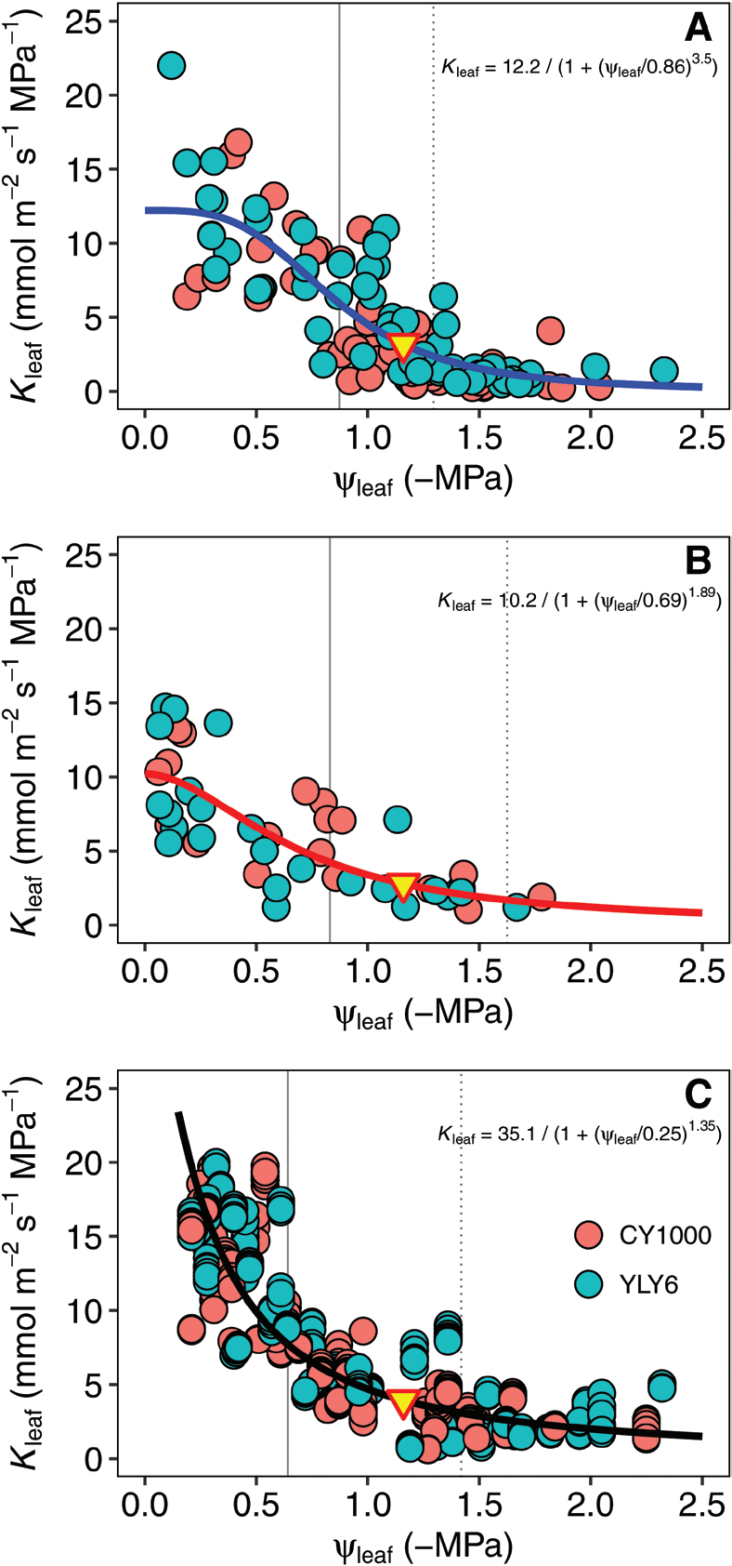
Vulnerability of leaf hydraulic conductance (*K*_leaf_) estimated by (A) the standard evaporative flux method (EFM), (B) the rehydration kinetic method, and (C) the gas exchange based EFM method. The vertical solid and dotted lines indicate the water potentials at 50% and 80% loss of *K*_leaf_, respectively. The triangle represents the turgor loss point (Table 1). Fitted lines are the best-fit functions selected using maximum likelihood.

The gas exchange and leaf hydraulic traits of rice were sensitive to short-term drought (Table 1; Figs 2 and 3). *A*, *g*_s_, and *g*_m_ declined exponentially with decreasing ψ_leaf_, with very similar patterns observed for the two genotypes (Fig. 2). Overall, the maximum *A*, *g*_s_, and *g*_m_ values were 19.30 µmol CO_2_ m^−2^ s^−1^, 0.31 mol H_2_O m^−2^ s^−1^, and 0.14 mol CO_2_ m^−2^ s^−1^, respectively, with slightly higher values in YLY6 than CY1000. To quantify the sensitivity of the gas exchange traits to leaf drying, we estimated the leaf water potential values at 50% and 80% loss of function (P_50_ and P_80_, respectively; Table 1; Fig. 2). The P_50_ values for *A*, *g*_s_, and *g*_m_ were –0.99 MPa, –0.93 MPa, and –1.03 MPa, respectively; however, the P_50_ of *J*_f_ was –1.99 MPa, which was lower than that of *A*.

**Table 1.** Pressure–volume, gas exchange, and leaf hydraulic vulnerability parameters of rice

Trait	CY1000	YLY6	Mean
SWC (g g^−1^)	2.31 ± 0.05	2.22 ± 0.06	2.27
π_0_ (MPa)	–0.67 ± 0.14	–0.83 ± 0.11	–0.75
π_tlp_ (MPa)	–1.13 ± 0.12	–1.19 ± 0.06	–1.16
*ɛ* (MPa)	8.41 ± 2.10	6.96 ± 0.79	7.69
*K*_max-RKM_ (mmol m^−2^ s^−1^ MPa^−1^)	8.02	9.25	8.47
*K*_max-EFM_ (mmol m^−2^ s^−1^ MPa^−1^)	12.2	11.73	11.9
*K*_max-licor_ (mmol m^−2^ s^−1^ MPa^−1^)	15.4	15.47	15.5
*A*_max_ (µmol CO_2_ m^−2^ s^−1^)	18.6	20.23	19.3
*g*_smax_ (mol H_2_O m^−2^ s^−1^)	0.29	0.33	0.31
*g*_mmax_ (mol CO_2_ m^−2^ s^−1^)	0.13	0.15	0.14
P_50-RKM_ (MPa)	–0.82	–0.85	–0.84
P_50-EFM_ (MPa)	–0.91	–0.82	–0.87
P_50-licor_ (MPa)	–0.64	–0.64	–0.64
P_50-*A*_ (MPa)	–0.93	–1.01	–0.99
P_50-*g*s_ (MPa)	–0.91	–0.94	–0.93
P_50-*g*m_ (MPa)	–0.99	–1.05	–1.03
P_50-*J*f_ (MPa)	–1.99	–1.98	–1.99

See Supplementary Table S1 for definitions of the parameters.

*K*_leaf_ vulnerability curves were determined using three independent methods (Fig. 3). Although the curves of the two genotypes were indistinguishable when estimated with the same method (Supplementary Table S2), the curves estimated using the three methods were different (Supplementary Table S3). The maximum *K*_leaf_ from the gas exchange based EFM method was 15.5 mmol m^−2^ s^−1^ MPa^−1^, almost twice as high as the 8.5 mmol m^−2^ s^−1^ MPa^−1^ estimated in the RKM method (Table 1). The P_50_ values of *K*_leaf_ were –0.84 MPa, –0.87 MPa, and –0.64 MPa for the RKM, EFM, and gas exchange based EFM methods, respectively. Moreover, the pressure–volume traits were similar in the two rice genotypes (Table 1), with average values for π_0_, π_tlp_, and *ɛ* of –0.75 MPa, –1.16 MPa, and 7.69 MPa, respectively.

### Photosynthetic limitation analysis

**Fig. 4.**
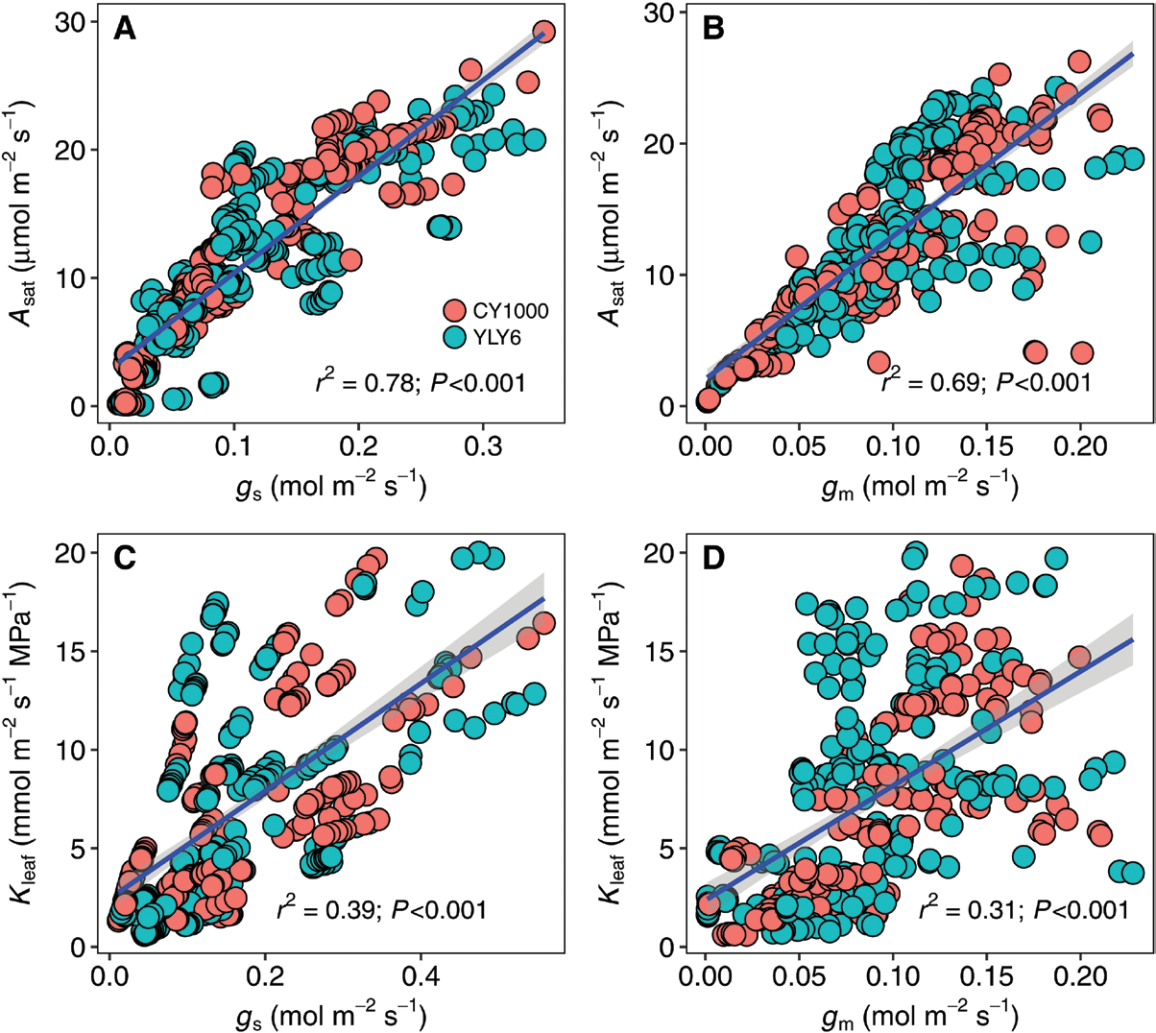
(A, B) Relationships between light-saturated photosynthetic rate (*A*) and stomatal conductance (*g*_s_) or mesophyll conductance to CO_2_ (*g*_m_). (C, D) Relationships between gas exchange based EFM estimation of leaf hydraulic conductance (*K*_leaf_) and *g*_s_ or *g*_m_.

**Fig. 5.**
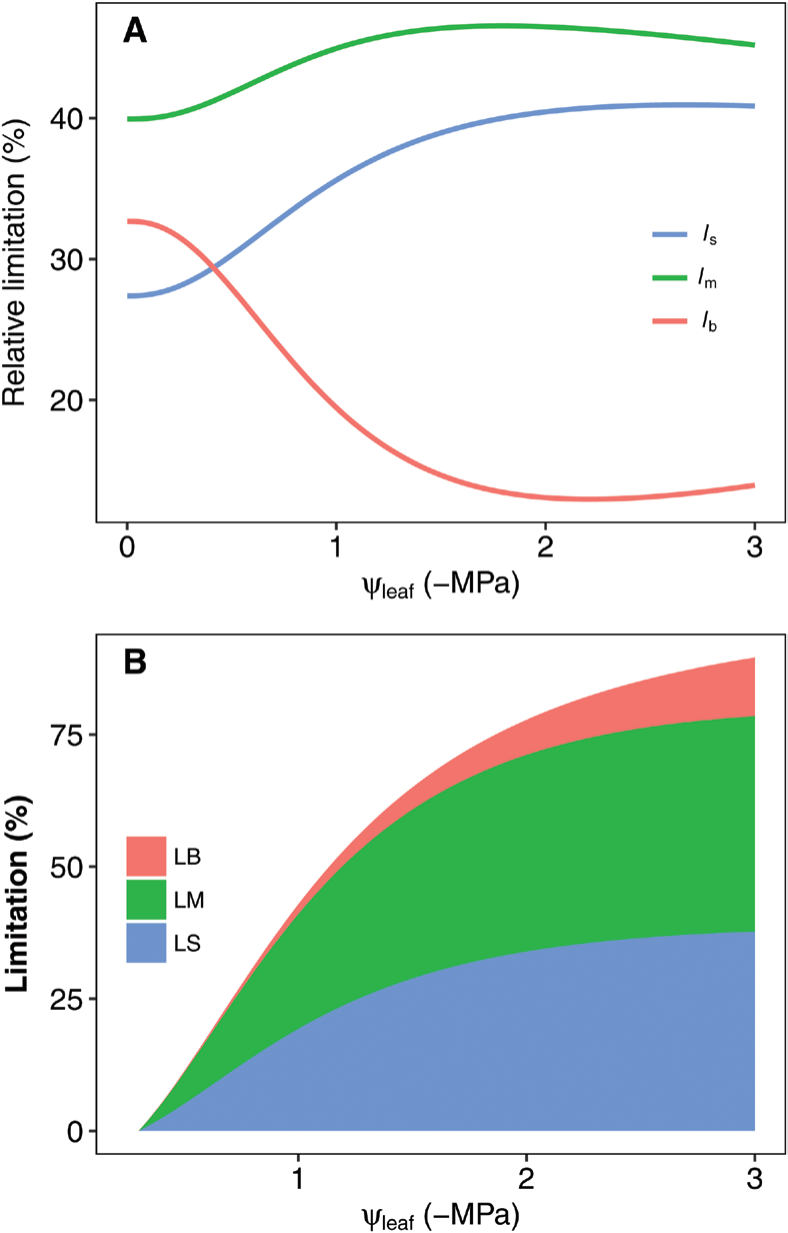
Effects of leaf water potential (ψ_leaf_) on (A) the distribution of the relative limits on photosynthesis caused by stomatal diffusion (*l*_s_), mesophyll diffusion (*l*_m_), and biochemistry (*l*_b_), and (B) the overall ψ_leaf_-dependent reduction in photosynthesis due to stomatal diffusion (LB), mesophyll diffusion (LM), and photosynthetic biochemistry (LB).

Both *g*_s_ (*r*^2^=0.78; *P*<0.001) and *g*_m_ (*r*^2^=0.69; *P*<0.001) were tightly correlated with *A* during the drought treatment (Fig. 4A, B). Close correlations were also observed between *K*_leaf_ and *g*_s_ (*r*^2^=0.39; *P*<0.001), and between *K*_leaf_ and *g*_m_ (*r*^2^=0.31; *P*<0.001). The impacts of drought on the relative stomatal (*l*_s_), mesophyll (*l*_m_), and biochemical (*l*_b_) limitations are shown in Fig. 5A. *g*_m_ was found to be the major limiting factor for photosynthesis in rice, as *l*_m_ contributed more than 40% of the relative limitation at any level of ψ_leaf_. As ψ_leaf_ decreased in response to soil drought, both *l*_s_ and *l*_m_ increased; however, *l*_b_ declined dramatically. Diffusion processes appear to have a prominent role in limiting photosynthesis during soil drying (Fig. 5B), with diffusion through stomata (LS) and mesophyll (LM) having the greatest effect. Overall, the diffusion limitation (LM+LS) reached ~50% at a ψ_leaf_ of –1.0 MPa, while the contribution of biochemistry (LB) to the limitation of photosynthesis was very small.

**Fig. 6.**
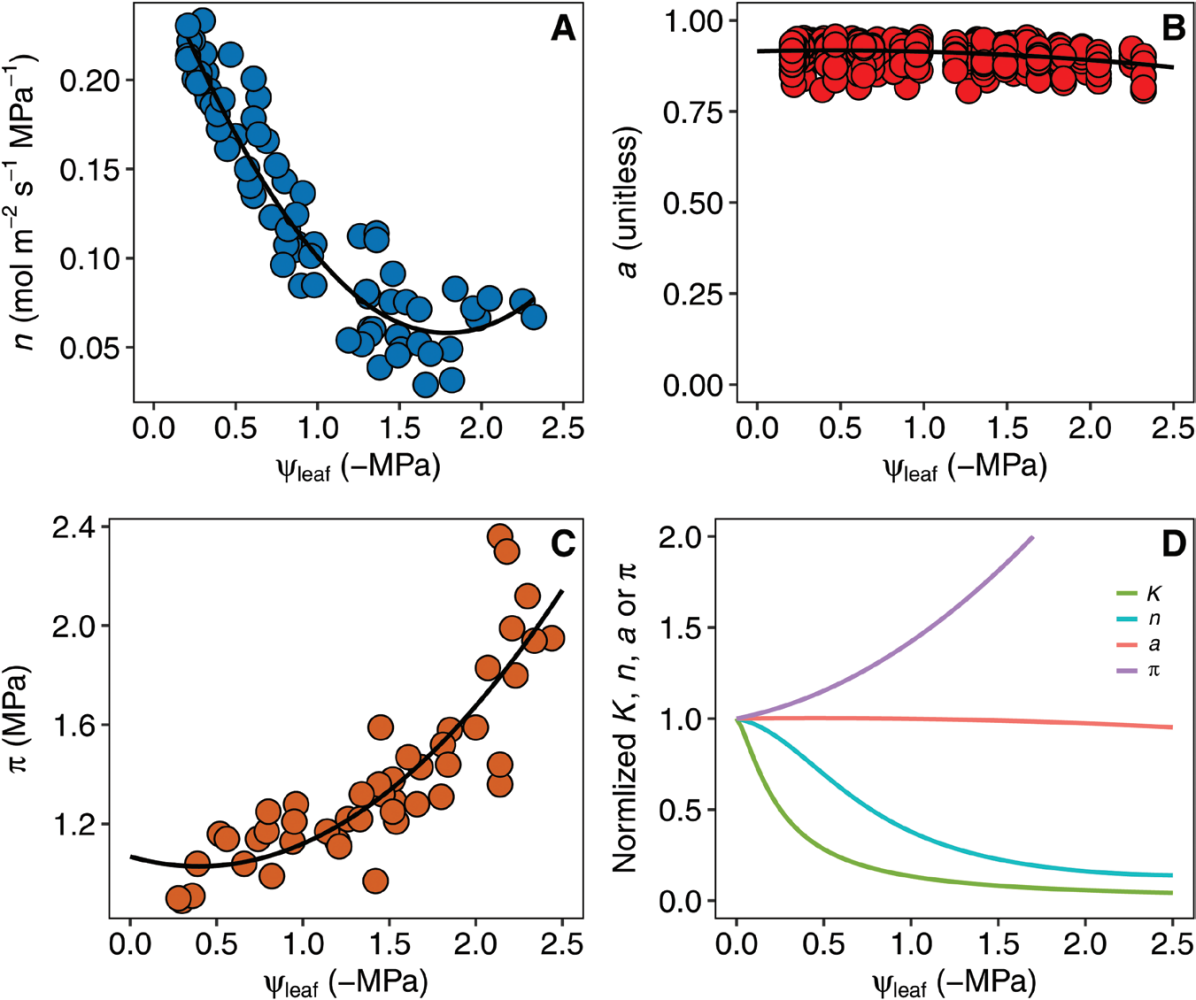
Responses of variables in the stomatal model to changes in leaf water potential (ψ_leaf_). (A) *n*, a turgor-independent parameter representing the effects of hormonal signals on the sensitivity of guard cell osmotic pressure to leaf turgor. (B) *a,* the relative concentration of adenosine triphosphate. (C) *π*, leaf osmotic pressure. (D) The parameters normalized by their values at a ψ_leaf_ of 0 MPa. *K*, gas exchange based EFM estimation of *K*_leaf_, as in Fig. 3C.

We fitted the stomatal model to our data and then partitioned the observed declines in *g*_s_ into contributions from each variable in the model (Fig. 6). The turgor-independent parameter, *n*, declined dramatically with leaf dehydration, with a 4-fold decrease as the leaf water potential decreased from 0 to –1.5 MPa during soil drought. The *a* parameter was quite stable during leaf dehydration; however, the leaf osmotic pressure, π, increased exponentially under drought.

## Discussion

In the present study, *A* declined under water stress, which resulted in a significant decrease in biomass accumulation. Photosynthesis in C_3_ plants such as rice is limited by *g*_s_, *g*_m_, and/or the biochemistry of photosynthesis itself, including the enzymes and metabolites involved in the process as well as components of the thylakoid electron transport chain (Flexas, 2016). Our analysis showed that the total relative limitation of photosynthesis by *g*_s_ and *g*_m_ was greater than 80% in rice when the ψ_leaf_ dropped to –1.0 MPa following soil drying (Fig. 5A). The results of the present study, as well as those of previous studies (Flexas *et al.*, 2002; Galle *et al.*, 2011; Galmés *et al.*, 2013), highlight a major role for CO_2_ diffusion in limiting *A* under conditions of water stress.

### The decrease in *g*_s_ can be largely explained by *K*_leaf_ vulnerability under drought

We found that the *g*_s_ of rice declined with decreases in the stem (ψ_stem_) and leaf (ψ_leaf_) water potentials under drought conditions (Fig. 2). This decrease in *g*_s_ during soil drought has been widely studied, although the mechanisms for this response remain unclear. Two major mechanisms that regulate stomatal closure under drought conditions have been suggested to involve hydraulic (Sperry *et al.*, 2002; Buckley, 2005; Brodribb & Cochard, 2009; Rodriguez-Dominguez *et al.*, 2016) or hormonal (Dodd, 2005; McAdam *et al.*, 2016) processes. Although hormonal signals were not measured in the present study, we quantified the responses to the hormonal and hydraulic signals of drought by modeling them (Fig. 6). Consistent with the findings of Rodriguez-Dominguez *et al.* (2016), we demonstrated that stomatal closure during drought can be largely explained by hydraulic signals, although hormonal signals also play a role in decreasing *g*_s_. Nevertheless, a recent study suggested that the drought-induced decline in *K*_leaf_ in isohydric grapevine (*Vitis vinifera*) genotypes is regulated by ABA accumulation (Coupel-Ledru *et al.*, 2017); thus, ABA could directly or indirectly regulate stomatal closure by decreasing *K*_leaf_. Future studies are required to clarify the direct and indirect impacts of ABA on stomatal closure under soil drought conditions.

The mechanisms of *K*_leaf_ decline during dehydration are still largely unknown. *K*_leaf_ consists of at least two components, the conductance within the xylem (*K*_x_) and the conductance through tissues outside the xylem (*K*_ox_); therefore, the decline of *K*_leaf_ during dehydration could potentially be caused by changes in either or both of these factors. Increases in xylem tension during dehydration can cause air bubbles to form in the xylem conduit pit (Brodribb *et al.*, 2016*a*; Brodribb *et al.*, 2016*b*; Skelton *et al.*, 2017), which decrease *K*_x_. When the tension in the xylem conduits exceeds the biomechanical resistance of the cell wall, the conduits collapse (Cochard *et al.*, 2004; Brodribb & Holbrook, 2005; Bouche *et al.*, 2016). *K*_x_ vulnerability cannot always fully explain the observed decline in *K*_leaf_; for instance, *K*_leaf_ can decline early, at high ψ_leaf_, before an embolism has been observed (Brodribb & Holbrook, 2006; Scoffoni *et al.*, 2012; Sack *et al.*, 2016). Indeed, some direct insights have challenged the major role for *K*_x_ vulnerability in driving *K*_leaf_ decline (Trifiló *et al.*, 2016; Scoffoni *et al.*, 2017*a*). In this study, we did not separate the contributions of *K*_x_ and *K*_ox_ to the decline in *K*_leaf_ during drought; however, Stiller *et al.* (2003) reported that the P_50_ of *K*_x_ in rice is approximately –2.0 MPa, which is far lower than that of the *K*_leaf_ observed here (Table 1), indicating that the decrease in *K*_leaf_ in rice during drought might be more closely connected to *K*_ox_ vulnerability. Water movement outside the xylem is complex and dynamic, involving apoplastic, symplastic, and transmembrane liquid flow paths and vapor diffusion within the intercellular airspaces (Buckley, 2015; Buckley *et al.*, 2015; Buckley *et al*., 2017). During leaf dehydration, cells may be less well connected to each other owing to changes in their shape and size caused by leaf shrinkage. Indeed, the initial slope of the vulnerability curve, before the turgor loss point, has been suggested to be more related to decreases in *K*_ox_ than *K*_x_ (Scoffoni *et al.*, 2014; Hernandez-Santana *et al.*, 2016). In this study, *K*_leaf_ decreased sharply before π_tlp_, suggesting that *K*_ox_ vulnerability played a major role in *K*_leaf_ decline. Moreover, the membrane permeability of the bundle sheath and mesophyll tissues has been suggested to influence *K*_leaf_, and this effect may be related to the activities of aquaporins (Laur & Hacke, 2014; Sade *et al.*, 2014*b*).

Currently, all methods for estimating *K*_leaf_ have limitations (both common and specific to each method) that require consideration when interpreting data (Flexas *et al.*, 2013). As shown in Fig. 3, the *K*_leaf_ vulnerability curve of rice is method dependent, despite the similar values observed between genotypes for any given method. The *K*_leaf_ vulnerability curve produced using EFM has a similar shape to the one generated using RKM (see the equations in Fig. 3 and statistics in Supplementary Table S2); however, the EFM method shows a much higher maximum *K*_leaf_ (*K*_max_; Table 1) value. Considering that the RKM measurement was performed in darkness, the difference may have been caused by light-dependent aquaporin activation (Cochard *et al.*, 2007; Scoffoni *et al.*, 2008). Indeed, we previously observed that rice *K*_leaf_ measured using EFM was strongly affected by light (Xiong *et al.*, 2018). In addition, the shape of the *K*_leaf_ vulnerability curve generated using the gas exchange based EFM method clearly differed from those produced using the other two methods, especially at high ψ_leaf_ values (>–0.5 MPa). One possible reason for these high *K*_leaf_ values at high ψ_leaf_ could be the imprecise method used to measure ψ_stem_. Although the leaves were wrapped and equilibrated overnight in this study, ψ_stem_ is technically challenging to measure precisely using pressure chambers in leaves that are close to full hydration.

### Responses of *g*_m_ to short-term soil drought

As reported for many species (Grassi & Magnani, 2005; Flexas *et al.*, 2006*a*; Warren, 2008; Flexas *et al.*, 2009; Galle *et al.*, 2009; Cano *et al.*, 2013), we observed that *g*_m_ in rice decreased with soil drought. Methodological problems exist in all currently available estimation techniques for *g*_m_ (Tholen *et al.*, 2012; Gu & Sun, 2014). One of the challenges for measuring *g*_m_ under drought conditions (low *g*_s_) is the accurate estimation of *C*_i_, because of the increasing relative contribution of *g*_cut_ to the overall leaf conductance, since the cuticular conductance for water is far greater than that for CO_2_. In this study, we carefully ruled out the effects of *g*_cut_ on *C*_i_. As it was not possible to estimate *g*_m_ under non-photorespiratory conditions using the variable *J* method, the effects of mitochondrial recycling of CO_2_ on *g*_m_ were not estimated here; however, the 3-fold decrease in *g*_m_ observed in this study is unlikely to have been caused by (photo)respiration alone. We therefore assume that the decrease in *g*_m_ values observed during drought was mostly due to the decline of *g*_m_*per se*.

The causes of the decrease in *g*_m_ during leaf dehydration are largely unknown, although *g*_m_ has been confirmed to be tightly correlated with mesophyll structure, membrane permeability, and the function of enzymes in the cytoplasm and chloroplast stroma (Flexas *et al.*, 2008; Evans *et al.*, 2009; Xiong *et al.*, 2015*a*; Xiong *et al.*, 2017). The two most important structural traits related to *g*_m_ are the cell wall thickness and the area of the chloroplast surface facing the intercellular airspace per unit leaf area (*S*_c_; Evans *et al.*, 2009; Tosens *et al.*, 2012; Tomás *et al.*, 2013; Tosens *et al.*, 2016; Xiong *et al.*, 2017). *S*_c_ is related to the mesophyll cells themselves, as well as to the shape of chloroplasts and the light-dependent arrangement of chloroplasts (Tholen *et al.*, 2008). During leaf dehydration, the chloroplasts may move to reduce photodamage to the photosystems, and thus potentially change the values of *S*_c_ (Tholen *et al.*, 2008). As for water transport outside the xylem, the decline of membrane permeability, mediated by aquaporins, is suggested to correspond with the decrease in *g*_m_ (Flexas *et al.*, 2006*b*; Perez-Martin *et al.*, 2014; Sade *et al.*, 2014*a*). The change in cell wall properties might be one of the reasons for the decline in *g*_m_ under drought, as water stress usually introduces changes in the bulk elastic modulus of the cell wall (Brodribb & Holbrook, 2003; Saito & Terashima, 2004; Guyot *et al.*, 2012), involving alteration of its biochemical composition and/or thickness. In addition, as shown in this study and previously (Théroux-Rancourt *et al.*, 2014; Théroux-Rancourt *et al.*, 2015), the *K*_leaf_, *A*, *g*_s_, and *g*_m_ vulnerability curves are almost always described as containing large measurement noise and/or high variability. Although estimation biases are inherently associated with all of the currently available techniques used to estimate *K*_leaf_ and *g*_m_, the large number of different leaves used to construct the curves may be responsible for the major sources of variability. Hence, developing new methods to construct vulnerability curves based on a single leaf is perhaps one way to reduce the estimation variability in the future.

### *K*_leaf_ vulnerability as a potential trigger for decline in *g*_s_ and *g*_m_

Correlations between *A*, *g*_s_, *g*_m_, and *K*_leaf_ have been widely observed in many species, in part because of the common pathways for CO_2_ diffusion and water transport within leaves, as well as between the leaf and atmosphere (Flexas *et al.*, 2013; Théroux-Rancourt et al., 2015; Xiong *et al.*, 2015*b*; Xiong *et al.*, 2018). To determine whether these traits truly influence each other, these correlations would need to be observed for plants grown in the same conditions and measured under variable environmental conditions. As shown in Fig. 4, rice grown in the same environment and exposed to short-term changes in soil water content displayed a positive correlation between *K*_leaf_ and both *g*_s_ and *g*_m_. Positive correlations between *g*_s_ and *K*_leaf_ across short-term environmental changes have been observed in many species (Théroux-Rancourt *et al.*, 2015; Gleason *et al.*, 2017; Xiong *et al.*, 2018); however, the positive correlation observed between *g*_m_ and *K*_leaf_ contradicts the findings of Loucos *et al.* (2017), who found no correlation between these traits in cotton (*Gossypium* sp.) measured under different light intensities. Although different species were used, the reason for this discrepancy is unclear. However, our results do support previous observations in grapevine (*Vitis* sp.) and poplar (*Populus* sp.) subjected to short-term soil drought (Ferrio *et al.*, 2012; Théroux-Rancourt *et al.*, 2014).

One of the novel findings of this study is the role of *K*_leaf_ vulnerability in triggering the decrease in *g*_s_ and *g*_m_. In general, changes in *A*, *g*_s_, and *g*_m_ in response to ψ_leaf_ were similar to the *K*_leaf_ vulnerability curves in rice; however, the P_50_ of *K*_leaf_ was higher than for *g*_s_ and *g*_m_ (Figs 2 and 3; Table 1). Our observations in rice disprove the previously proposed hypothesis, which suggested that stomata close early to reduce xylem tension and thus prevent plant hydraulic dysfunction (Cochard *et al.*, 2004; Brodribb & Holbrook, 2006; Hochberg *et al.*, 2017). As discussed above, *K*_leaf_ vulnerability might be largely determined by the non-xylem water movement pathways, and thus be influenced directly by the hydraulic effects that also trigger stomatal closure (Brodribb & Holbrook, 2003; Guyot *et al.*, 2012). Indeed, a recent study showed that stomatal closure under drought is induced by hydraulic signals but maintained by ABA (Tombesi *et al.*, 2015).Changes in hormone levels and/or leaf structural properties potentially decrease *g*_m_. Interestingly, accumulation of ABA during drought conditions has been reported to decrease *g*_m_ significantly (Mizokami *et al.*, 2015); moreover, a slight increase in the leaf ABA level is enough to decrease *g*_s_, but decreases in *g*_m_ require higher leaf levels of ABA (Mizokami *et al.*, 2015). The observation that *g*_s_ is more sensitive to drought than *g*_m_ may relate to the accumulation of ABA in leaves.

### Modification of leaf anatomy facilitates the acclimation of leaf physiology to long-term drought

The acclimation of leaf anatomy and physiology to long-term drought was found to be coordinated in rice (Fig. 1). The LA and LW of the two rice genotypes displayed coordinated acclimation to drought, with the decrease in LA largely resulting from the narrowing of the leaf. Interestingly, a previous study found that grass species with naturally narrow leaves have high physiological drought tolerance (Craine *et al.*, 2012). The decrease in LW is also associated with an increase in leaf vein density, which could result from the declining LW and/or increasing vein numbers. For instance, a perfectly coordinated acclimation of vein density and LW would suggest that vein spacing is determined passively by differences in leaf expansion. In this study, the major vein (VLA_major_) and minor vein densities(VLA_minor_) increased to different degrees under drought, suggesting that the increased leaf vein density is regulated both passively and actively in rice (Fig. 1). Moreover, the genotype-dependent differences in leaf vein density changes under drought may underpin the different drought tolerances of the two genotypes studied. The acclimation of the physiological traits to long-term drought was genotype dependent, providing further evidence that modification of leaf vein density facilitates the physiological acclimation to drought in rice. Higher leaf vein densities in drought-acclimated leaves have a higher hydraulic capacity, and thus assimilate higher quantities of carbon. Vein density is closely related to *K*_leaf_ because greater vein densities, especially of the minor veins, are associated with higher *K*_ox_ and *K*_x_ values (Buckley *et al.*, 2015). In the present study, the responses of *g*_m_ and *g*_cut_ were also genotype dependent, suggesting that the mesophyll and epidermal tissues are also responsive to physiological acclimation in rice. However, we could not evaluate the effects of drought-induced anatomical and physiological changes on drought tolerance capacity because we did not construct pressure–volume curves and *K*_leaf_ vulnerability curves after drought treatment. Future research should focus on the effects of anatomical and physiological changes on drought tolerance.

In conclusion, these results provide new evidence that *K*_leaf_ and gas exchange are coordinated under drought conditions. Photosynthesis under drought conditions is primarily limited by *g*_s_ and *g*_m_, and the decreased *g*_s_ was mainly determined by the decline in *K*_leaf_, although it was also related to drought-induced hormonal signals. The decreased *g*_m_ and *K*_leaf_ are likely related to the changes in leaf anatomy and membrane permeability caused by drought.

## Supplementary data

Supplementary data are available at *JXB* online.

Table S1. List of mathematical parameters and their units of measurement.

Table S2. Two-sample Kolmogorov-Smirnov test results in comparing *K*_leaf_ vulnerability of two rice genotypes.

Table S3. Two-sample Kolmogorov-Smirnov test results comparing *K*_leaf_ vulnerability methods.

Fig. S1. Climate information during the experiment (2017).

Supplementary Tables and Figure
