# Supplementary material for: Leaf hydraulic vulnerability triggers the decline in stomatal and mesophyll conductance during drought in rice
Source: J Exp Bot. 2018 May 18;69(16):4033–45. doi: 10.1093/jxb/ery188 (PMC6054168; doi:10.1093/jxb/ery188)
Supplement: Supplementary Tables and Figure [file ery188_suppl_supplementary_tables-s1-s3_figure-s1.pdf]

Leaf hydraulic vulnerability triggers the decline in stomatal and mesophyll conductance during drought in rice (*Oryza sativa*)

Xiaoxiao Wang[1#], Tingting Du[1#], Jianliang Huang[1], Shaobing Peng[1] and Dongliang Xiong[1,2*]

[*]Corresponding author

Email: dlxiong@mail.hzau.edu.cn

dlxiong@ucdavis.edu

Supplementary data

Table S1. List of mathematical parameters and their units of measurement

**Description**	**Symbol**	**Unit**
Bulk leaf osmotic pressure	π	MPa
Biomass	BM	g $plant^{-1}$
Contribution of biochemistry to a change in photosynthesis	LB	%
Contribution of g_m to a change in photosynthesis	LM	%
Contribution of g_s to a change in photosynthesis	LS	%
Cuticular conductance	g_{cut}	mol m^{-2} s^{-1}
Electron transport rates	J_f	μmol m^{-2} s^{-1}
Leaf area	LA	cm^2
Leaf width	LW	cm
Leaf mass per area	LMA	g m^{-2}
Leaf hydraulic conductance (maximum)	K_{leaf} (K_{max})	mmol m^{-2} s^{-1} MPa^{-1}
Leaf hydraulic conductance based on li-cor transpiration rate	K_{leaf_licor}	mmol m^{-2} s^{-1} MPa^{-1}
Leaf hydraulic conductance measured by evaporative flux method	K_{leaf_EFM}	mmol m^{-2} s^{-1} MPa^{-1}
Leaf hydraulic conductance measured by rehydration kinetic method	K_{leaf_RKM}	mmol m^{-2} s^{-1} MPa^{-1}
Leaf osmotic potential	$\Psi_{osmotic}$	MPa
Leaf water potential	ψ_{leaf}	MPa
Light-saturated photosynthetic rate (maximum)	A (A_{max})	μmol m^{-2} s^{-1}
Leaf vein density	VLA	mm mm^{-2}
Major vein density	VLA_{major}	mm mm^{-2}
Minor vein density	VLA_{minor}	mm mm^{-2}
Mesophyll conductance (maximum)	g_m (g_{mmax})	mol m^{-2} s^{-1}
Modulus of elasticity	ε	MPa
Osmotic potential at full turgor	π_0	MPa
Ratio of water mass to leaf dry mass in a fully hydrated leaf	SWC	g g^{-1}
Relative limitation of biochemistry to A	l_b	%
Relative limitation of g_m to A	l_m	%
Relative limitation of g_s to A	l_s	%
Relative adenosine triphosphate (ATP) concentration	a	unitless
Stem water potential	ψ_{stem}	MPa
Stomatal conductance (maximum)	g_s (g_{smax})	mol m^{-2} s^{-1}
Turgor-independent parameter	n	mmol m^{-2} s^{-1} MPa^{-1}
Turgor loss point	π_{tlp}	MPa
Water potential at J_f reduces 50% (80%)	P_{50_Jf} (P_{80_Jf})	MPa
Water potential at K_{leaf_EFM} reduces 50% (80%)	P_{50-EFM} (P_{80_EFM})	MPa
Water potential at K_{leaf_licor} reduces 50% (80%)	$P_{50-licor}$ (P_{80_Licor})	MPa
Water potential at K_{leaf_RKM} reduces 50% (80%)	P_{50-RKM} (P_{80_RKM})	MPa
Water potential at g_m reduces 50% (80%)	P_{50_gm} (P_{80_gm})	MPa
Water potential at A reduces 50% (80%)	P_{50_A} (P_{80_A})	MPa
Water potential at g_s reduces 50% (80%)	P_{50_gs} (P_{80_gs})	MPa

Table S2 Two-sample Kolmogorov-Smirnov test results in comparing K_{leaf} vulnerability of two rice genotypes. D, Kolmogorov-Smirnov maximum distance

Method	D	*p*
K_{leaf_EFM}	0.12	0.104
K_{leaf_RKM}	0.04	0.916
K_{leaf_licor}	0.06	0.826

Table S3 Two-sample Kolmogorov-Smirnov test results in comparing K_{leaf} vulnerability methods. D, Kolmogorov-Smirnov maximum distance.

	K_{leaf_licor}	K_{leaf_RKM}
K_{leaf_EFM}	0.19 (0.108)	0.31 (0.003)
K_{leaf_RKM}	0.28 (<0.001)	

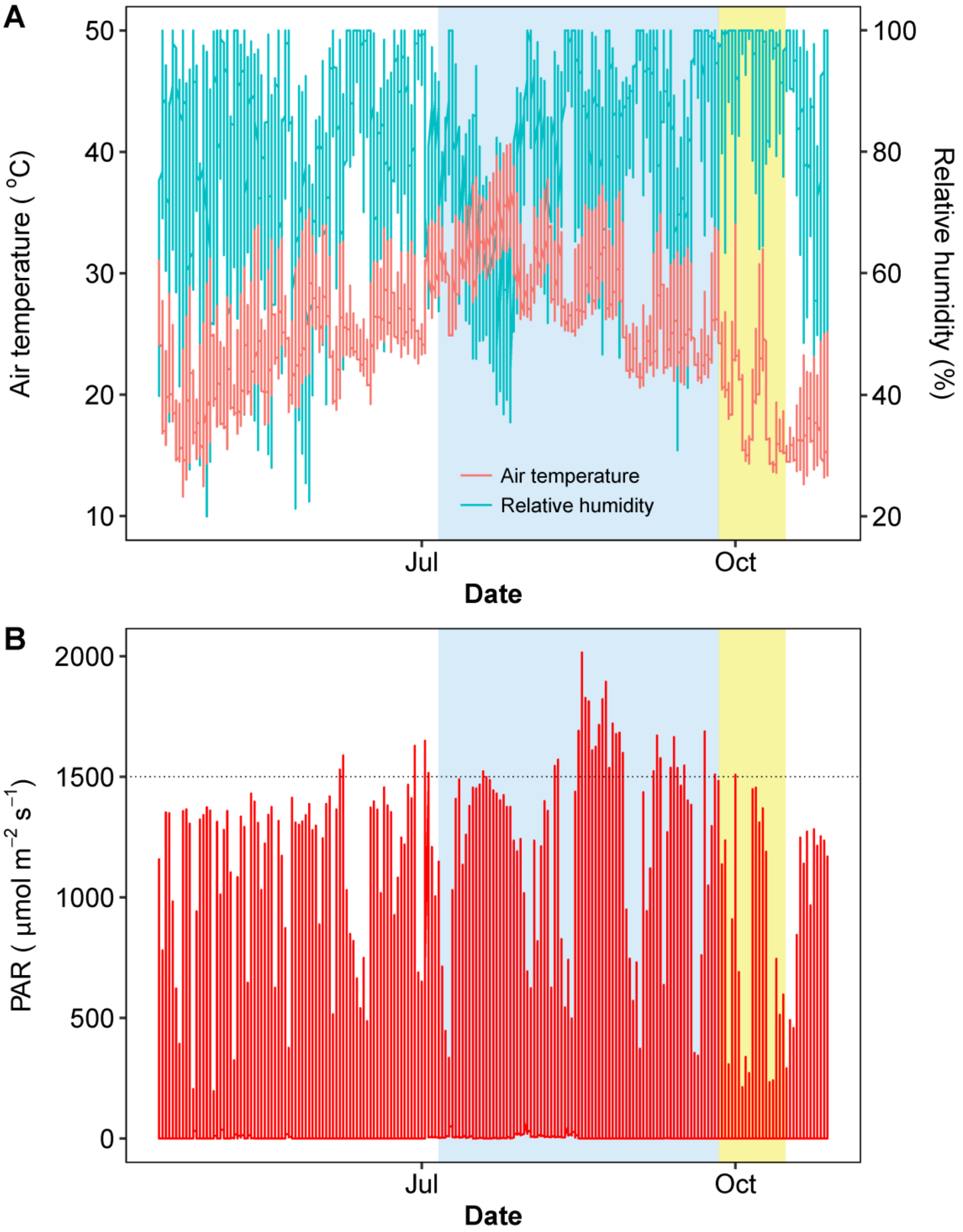

Figure S1. Climate information (2017). The shaded area indicates the whole experimental period and the yellow area indicates the drought treatment duration. Data were recorded every 10 minutes.
